# New data on spiders (Arachnida, Araneae) of the Arabian Peninsula and the Levant, with new species and records

**DOI:** 10.3897/zookeys.1276.186138

**Published:** 2026-04-03

**Authors:** Alireza Zamani, Yuri M. Marusik, Mostafa R. Sharaf, Khafiya Al Ketbi, Tamás Szűts

**Affiliations:** 1 Zoological Museum, Department of Biodiversity Sciences, University of Turku, Turku, Finland Department of Zoology & Entomology, University of the Free State Bloemfontein South Africa; 2 Institute for Biological Problems of the North, FEB RAS, Portovaya Str. 18, Magadan, Russia Institute for Biological Problems of the North, FEB RAS Magadan Russia; 3 Department of Zoology & Entomology, University of the Free State, Bloemfontein, South Africa Department of Zoology, University of Veterinary Medicine Budapest Budapest Hungary; 4 Al Dhaid Wildlife Museum, Environment and Protected Areas Authority (EPAA), Sharjah, United Arab Emirates Department of Biodiversity Sciences, University of Turku Turku Finland; 5 Department of Zoology, University of Veterinary Medicine Budapest, Budapest, Hungary Al Dhaid Wildlife Museum, Environment and Protected Areas Authority (EPAA) Sharjah United Arab Emirates

**Keywords:** Kuwait, Lebanon, Middle East, Saudi Arabia, taxonomy, United Arab Emirates

## Abstract

Based on several small collections, new taxonomic and faunistic data on spiders of the United Arab Emirates (UAE), Saudi Arabia, Kuwait, and Lebanon are presented. Six species are described as new to science: *Dorceus saif* Szűts & Zamani, **sp. nov**. (♂; Saudi Arabia), *Loureedia melanconi* Szűts & Zamani, **sp. nov**. (♂; Saudi Arabia) (both Eresidae), *Leptopilos hajarensis* Zamani & Marusik, **sp. nov**. (♀; UAE), *Synaphosus dulcicola* Zamani & Marusik, **sp. nov**. (♀; UAE) (both Gnaphosidae), *Arctosa formosa* Zamani & Marusik, **sp. nov**. (♂♀; UAE) (Lycosidae), and *Prodidomus emiratus* Zamani & Marusik, **sp. nov**. (♀; UAE) (Prodidomidae). In addition, the previously unknown male of *Trichothyse golan* (Levy, 1999) (Gnaphosidae) is illustrated and described. Finally, one new family record, 16 new generic records and 22 new species records are documented, and the previous record of *Sahastata infuscata* (Kulczyński, 1901) (Filistatidae) from Yemen is rejected.

## Introduction

Spiders are understudied in West Asia, particularly in the Arabian Peninsula. Although the faunas of Yemen, Oman, and the United Arab Emirates (UAE) have received some historical (e.g., Pocock 1903; Denis 1953) or more recent (e.g., Saaristo and van Harten 2006; Grasshoff and van Harten 2007; Zamani 2018; Zamani et al. 2025b) taxonomic attention, much of the latter owing to the “Zoological Survey of Arabia” (see Büttiker 1987), the faunas of Saudi Arabia, Kuwait, Bahrain, and Qatar remain either very poorly known or entirely undocumented. Among these countries, checklists have only been published for Saudi Arabia (El-Hennawy 2014) and the UAE (Feulner and Roobas 2015). Most other countries in the region also remain poorly studied and lack checklists. Lebanon and Syria, for example, are known almost entirely from material treated in historical publications (e.g., Pickard-Cambridge 1872; Denis 1955), with only a few studies focusing specifically on their faunas (e.g., Assi 2013). Recently, we had the opportunity to examine several small collections from the UAE, Saudi Arabia, Kuwait, and Lebanon, which yielded several new species and distributional records. The results of this work are presented in this paper.

## Materials and methods

### Collection sites and habitats

Material from Lebanon was collected using pitfall traps and beating nets in two montane woodlands dominated by mixed broadleaf trees. Material from Kuwait was collected using beating nets in an arid area with low sand and gravel plains, except for *Latrodectus dahli* Levi, 1959, which is included here on the basis of museum material. Material from Saudi Arabia was obtained through opportunistic hand-collecting at two sites: the eresids were collected near Tanajib Airport in a coastal desert with low sand and gravel plains, whereas the filistatid was collected in a village situated in a semi-arid, rocky area with sparse xerophytic shrubs and scattered drought-tolerant trees.

**Figure 1.**
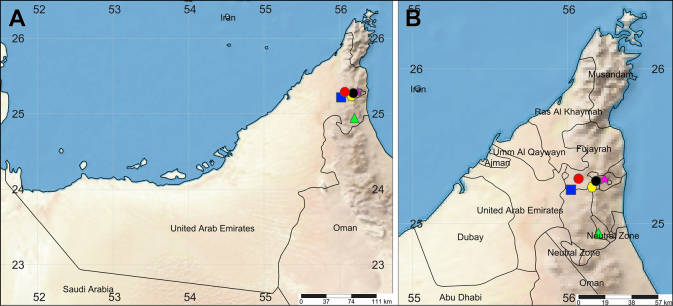
Collection localities in the UAE. **A**. Map of the entire country; **B**. Map of the northern region in detail. Yellow hexagon–Masafi; red circle–Masafi-Al Dhaid Rd.; black circle–Daftah; blue sqaure–Kedra; pink star–Wadi Shees; green triangle–Wadi Al Helo.

**Figure 2.**
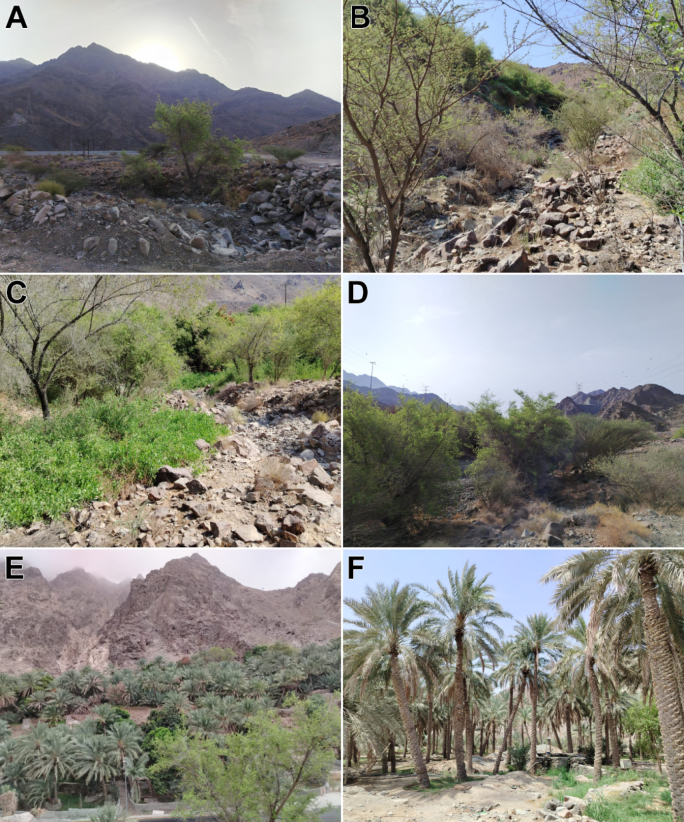
Habitats at the collection localities in the UAE. **A**. Masafi; **B**. Daftah; **C**. Kedra; **D**. Masafi-Al Dhaid Rd.; **E**. Wadi Shees; **F**. Wadi Al Helo. Photos: MRS.

In the UAE, the mountain environment occupies most of Fujairah, much of Ras Al Khaimah, parts of Sharjah, and the Dubai and Ajman enclaves of Hatta and Masfut, respectively (Feulner 2024). The Emirati specimens examined here were collected using various methods, including direct hand collecting and pitfall and yellow pan traps for terrestrial species, and beating sheets for arboreal species. Sampling was conducted at several localities in and around the Hajar Mountains (Fig. 1A, B), as follows:

- Masafi (Fig. 2A), located between Fujairah and Ras Al Khaimah, is known for its exceptionally high biodiversity: more than half of the UAE's native plant species occur within less than five percent of the country's land area here (Böer and Chaudhary 1999; Feulner 2024). Additional specimens were collected along the road between Masafi and Al Dhaid (Fig. 2D).
- Daftah (Fig. 2B), a village near the Ras Al Khaimah-Fujairah border at ~629 m elevation, has vegetation influenced by both altitude and the seasonal flow of Wadi Ham. Plant communities are richer than in nearby coastal plains, comprising drought-tolerant species along with those adapted to the wadi channel (Deil and Müller-Hohenstein 1996).
- Kedra (Fig. 2C), in Ras Al Khaimah, comprises mountains, plains, and wadis with drought-tolerant vegetation, dominated by trees (*Acacia tortilis*, *A. ehrenbergiana*, *Prosopis cineraria*, *Ziziphus spina-christi*) and shrubs (*Calotropis procera*, *Euphorbia larica*, *Fagonia indica*, *Leptadenia pyrotechnica*) on rocky slopes (Feulner 2024).
- Wadi Shees (Fig. 2E), located near Khorfakkan in the Hajar Mountains, supports denser and more diverse vegetation than adjacent desert areas, owing to freshwater sources in its valleys (Feulner 2024).
- Wadi Al Helo (Fig. 2F) in Sharjah is a protected area within the Hajar Mountains, particularly known for its abundance of *Z. spina-christi* (Feulner 2024). The presence of perennial water makes the wadi noticeably greener than much of the surrounding areas.

### Technical data and morphological examination

Habitus of the two eresids was photographed using a Tucsen MIchrome digital camera mounted on a Nikon SMZ1000 stereomicroscope, while images of the palps were taken with a Tucsen TrueChrome Metrics digital camera on a Nikon Eclipse E200 compound microscope, both in the Department of Zoology, University of Veterinary Medicine Budapest. Photographs of the remaining specimens were obtained using an Olympus Camedia E-520 camera attached to an Olympus SZX16 stereomicroscope at the Zoological Museum of the University of Turku. Illustrations of endogynes were made after digesting tissues off in a 10% KOH aqueous solution. Digital images of different focal planes were stacked with Helicon Focus™ 8.1.1. Body measurements exclude the chelicerae and spinnerets. Leg segments were measured on the dorsal side. Measurements of palp and legs are listed as: total (femur, patella, tibia, metatarsus [absent in palp], tarsus). All measurements are given in millimeters. Species distributions follow WSC (2026), with minor modifications in some cases. The distribution maps (Fig. 1A, B) were created using SimpleMappr (Shorthouse 2010).

### Depositories

**DWLC** Al Dhaid Wildlife Museum, Environment and Protected Areas Authority (EPAA), Sharjah (M.R. Sharaf);

**HNHM** Hungarian Natural History Museum, Budapest (E. Lazányi);

**SMNHTAU** The Steinhardt Museum of Natural History, Tel Aviv (S. Zonstein);

**SMNS** Staatliches Museum für Naturkunde Stuttgart (I. Wendt);

**ZMUT** Zoological Museum of the University of Turku (V. Vahtera).

### Molecular procedures and phylogenetic analysis

DNA extraction, PCR, and sequencing were carried out by Krisztián Szabó (Molecular Ecology Research Group, Department of Zoology, University of Veterinary Medicine Budapest) and are described in detail in Zamani et al. (2025a). All analyses were performed on the NGPhylogeny server (Lemoine et al. 2019). The matrix of Szűts et al. (2023) was supplemented with five additional sequences. Sequences were aligned with MAFFT (Katoh and Toh 2008), the alignment was processed in BGME (Criscuolo and Gribaldo 2010), model selection was conducted with SMS (Lefort et al. 2017), and maximum-likelihood analyses were performed in PhyML (Guindon et al. 2010) on the NGPhylogeny server (Lemoine et al. 2019). Bootstrap values were calculated following Lemoine et al. (2019), and the resulting tree was visualized and edited in iTOL (Letunic and Bork 2024).

## Results

### Taxonomy

**Family Dictynidae O. Pickard-Cambridge, 1871**

#### Nigma conducens

Taxon classificationAnimaliaAraneaeDictynidae

(O. Pickard-Cambridge, 1876)

E8017612-3D99-54E0-816F-B05462977811

##### Material.

UAE: Fujairah: • 3♀ (DWLC), Al Dhaid–Masafi Rd., 25°17'42.0"N, 56°04'33.6"E, 298 m, 10.9.2025 (M.R. Sharaf).

##### Distribution.

Previously known only from North Africa (Morocco to Egypt). New genus record for the UAE, with the present material representing the easternmost known record of the species.

###### Family Eresidae C.L. Koch, 1845

#### Dorceus saif

Taxon classificationAnimaliaAraneaeEresidae

Szűts & Zamani, sp. nov.

DFDF9D60-0ECB-5244-A648-87890212A161

https://zoobank.org/C9658AC5-7938-4122-B8D7-B2D8A9396E57

Fig. 3A–G

##### Type material.

***Holotype*** • ♂ (HNHM11596), Saudi Arabia: Eastern Prov.: As Saffaniyah, Saudi Aramco Tanajib Airport, 27°52'N, 48°46'E, 10.6.2021 (C.J. Melancon).

##### Diagnosis.

**Figure 3.**
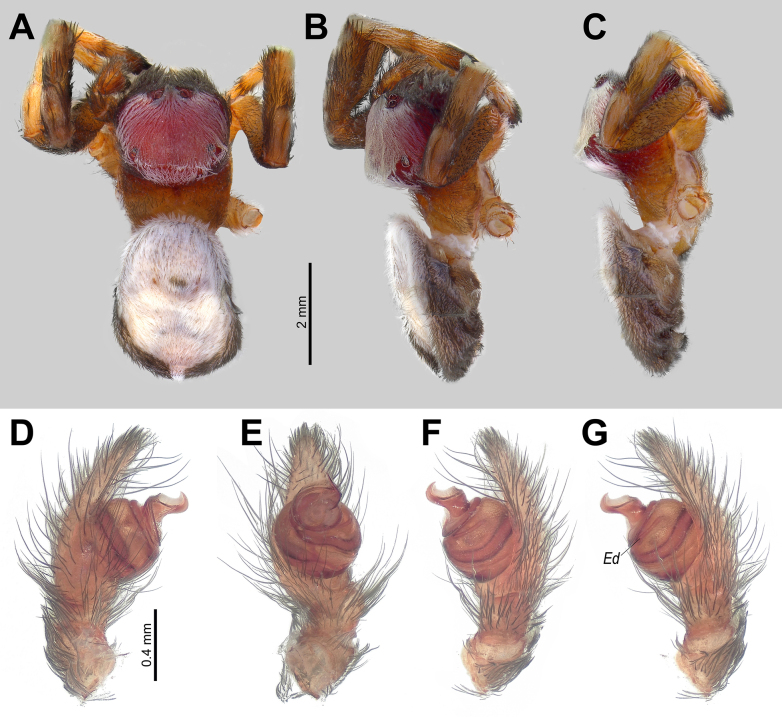
*Dorceus saif* sp. nov., male. **A–C**. Habitus, dorsal, dorsolateral, and lateral views; **D, E**. Palp, prolateral and ventral views; **F, G**. Ditto, varying retrolateral views. Abbreviation: *Ed* = ejaculatory duct.

The male of this species can be distinguished from most congeners by its long conductor blade. It resembles *D. trianguliceps* Simon, 1911 in having a strongly curved, broad conductor tip, but differs in the blade being more protruding and in the ejaculatory duct diverging from the base of the conductor (vs parallel; cf. Fig. 3F, G vs El-Hennawy 2002: fig. 14). The coloration pattern is also diagnostic: the cephalic area is entirely covered with white setae, whereas the thoracic area lacks them (Fig. 3A–C), and the abdominal dorsum is white (Fig. 3A).

##### Description.

**Male**. Habitus as in Fig. 3A–C. Total length 5.75. Carapace 3.20 long, 2.20 wide, 1.80 high. Carapace integument yellow, covered entirely with white setae in pars cephalica, reaching down to middle eyes; chelicerae and frontal side of carapace below middle eyes covered densely with thick black setae (Fig. 3B); pars thoracica with sparse black slender setae; labium, sternum, and maxillae pale brown. Femora and proximal end of tibiae with mainly black setae (few white setae on proximal part), other parts of legs with white setae. Abdomen white dorsally, dark grey laterally and ventrally. Spinnerets dark grey. Measurements of legs: I: 6.90 (2.15, 1.20, 1.25, 1.50, 0.80); II: 6.65 (2.00, 1.20, 1.15, 1.60, 0.70); III: 5.75 (1.95, 0.80, 1.00, 1.20, 0.80); IV: 7.80 (2.60, 1.10, 1.65, 1.55, 0.90).

Palp as in Fig. 3D–G; spermophor slightly bent medially (Fig. 3F, G); ejaculatory duct (*Ed*) diverging from conductor base (Fig. 3F); conductor long, curved blade-shaped in lateral view.

**Female**. Unknown.

##### Distribution.

Known only from the type locality in the Eastern Province, northeastern Saudi Arabia. This is the first record of *Dorceus* C.L. Koch, 1846 from this country and the easternmost known record of the genus.

##### Etymology.

The specific epithet is an Arabic word meaning “sword,” referring to the shape of the conductor in lateral view.

#### Loureedia melanconi

Taxon classificationAnimaliaAraneaeEresidae

Szűts & Zamani, sp. nov.

877A5FB9-3240-578B-AE18-2B7DD0F0450A

https://zoobank.org/A765907E-ECD7-4877-A123-348E2C6547DA

Fig. 4A–F

##### Type material.

***Holotype*** • ♂ (HNHM11521), Saudi Arabia: Eastern Prov.: As Saffaniyah, Saudi Aramco Tanajib Airport, 27°52'N, 48°46'E, 17.11.2021 (C.J. Melancon).

##### Diagnosis.

**Figure 4.**
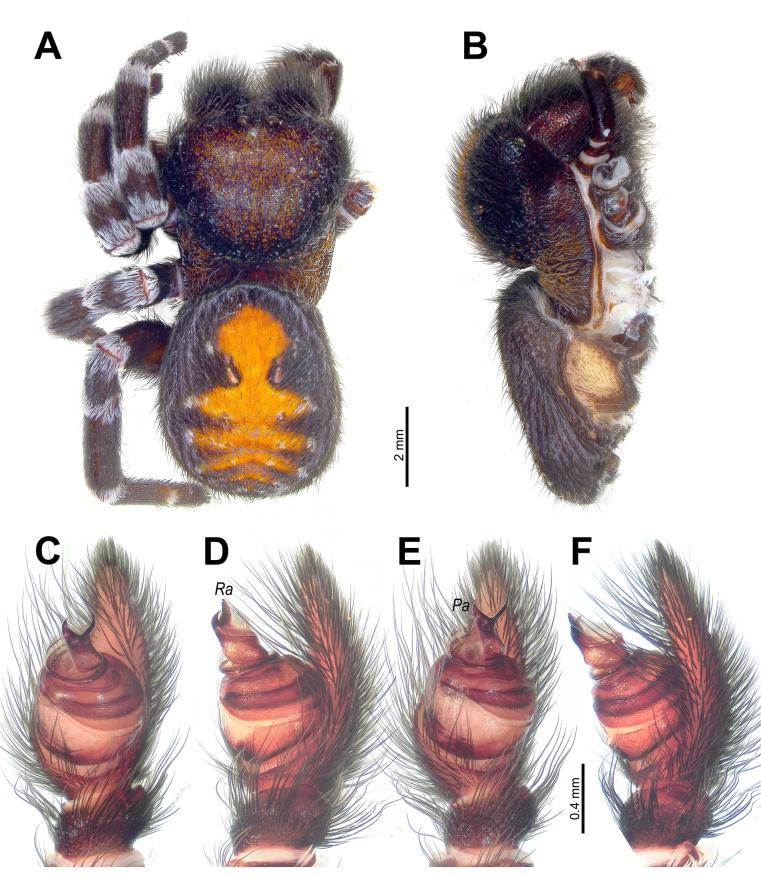
*Loureedia melanconi* sp. nov., male. **A, B**. Habitus, dorsal and lateral views; **C, E**. Palp, varying ventral views; **D, F**. Ditto, varying retrolateral views. Abbreviations: *Pa* = prolateral arm of the conductor; *Ra* = retrolateral arm of the conductor.

The prolateral arm of the conductor (*Pa*) is strongly bent, resembling that of *L. phoenixi* Zamani & Marusik, 2020 and *L. jerbae* (El-Hennawy, 2005) (cf. Fig. 4D vs Szűts et al. 2023: fig. 6A, B). It can be distinguished from *L. phoenixi* by the larger retrolateral arm of the conductor (*Ra*; Fig. 4F), and from *L. jerbae* by the apical curvature of the retrolateral conductor margin (vs straight), as well as by the blunt tip of the retrolateral arm of the conductor (vs pointed). The male also differs from *L. phoenixi* in body coloration, particularly in lacking large white dorsal abdominal spots (cf. Fig. 4A vs Zamani and Marusik 2020: fig. 1a–f). The general coloration of the species is very similar to that of *L. maroccana* Gál, Kovács, Bagyó, Vári & Prazsák, 2017 and *L. jerbae*, but the patellae bear broad stripes on both ends (~20% of the segment length covered with black setae, as in *L. phoenixi*), whereas *L. maroccana* and *L. jerbae* have only thin white stripes in these areas (cf. Fig. 4A vs Szűts et al. 2023: fig. 2B, D).

##### Description.

**Male**. Habitus as in Fig. 4A, B. Total length 12.40. Carapace 6.55 long, 5.25 wide, 3.80 high. Carapace, sternum, labium, chelicerae, and maxillae dark walnut brown with reddish shine. Carapace anteriorly densely covered with long black setae; pars cephalica covered with red scales, pars thoracica covered with red and scattered white setae. Legs covered with thin black setae, with distinct thick, dense bands of white setae at all joints. Abdomen covered with black setae, dorsally with crimson red longitudinal foliate pattern bearing white spots at its lateral extensions; anteriormost part of median globular pattern trilobed, lateral lobes white and anterior lobe crimson red. Spinnerets black. Measurements of legs: I: 12.40 (3.85, 2.20, 2.75, 2.25, 1.35); II: 11.65 (3.80, 1.95, 2.30, 2.25, 1.35); III: 10.40 (3.45, 2.05, 2.10, 1.80, 1.00); IV: 12.75 (4.15, 2.15, 3.05, 2.30, 1.10).

Palp as in Fig. 4C–F; stem of conductor ~1.5× longer than wide, with straight prolateral margin forming right angle with posterior margin, and slightly curved retrolateral margin; prolateral arm of conductor (*Pa*) sharp-tipped and slightly shorter than blunt-tipped retrolateral one (*Ra*).

**Female**. Unknown.

##### Remarks.

**Figure 5.**
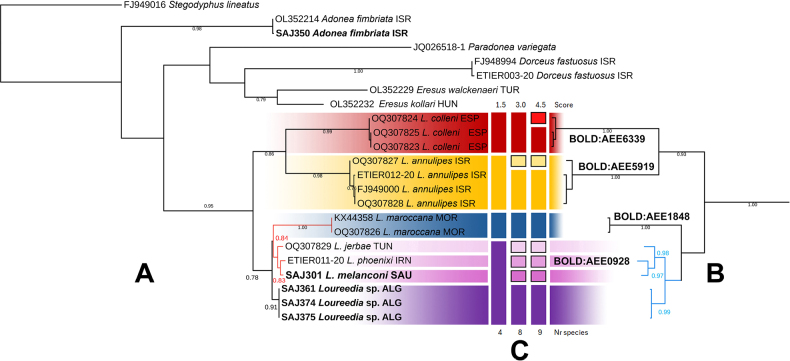
Phylogenetic hypotheses and species delimitations for *Loureedia* species based on the COI marker. **A**. Maximum likelihood (GTR+G+I model); **B**. Bayesian inference; **C**. ASAP species delimitation analysis. Sequences generated in this study are indicated in bold.

Molecular analyses were conducted to evaluate the phylogenetic relationships of the species. These analyses consistently recovered the *L. jerbae*-*L. phoenixi*-*L. melanconi* clade, with a closer relationship between the latter two, with another cryptic species from Algeria (Fig. 5A). The phylogenetic analyses produced two alternative topologies: the maximum-likelihood analysis placed the *L. jerbae*-*L. phoenixi*-*L. melanconi* clade as sister to *L. maroccana*, whereas the Bayesian analysis suggested a sister relationship to the cryptic Algerian species (Fig. 5B). However, none of these groupings received strong support.

The three best-scoring ASAP results were largely congruent. Two supported the *L. jerbae*-*L. phoenixi*-*L. melanconi* clade as three species (and also indicated partitioning within *L. annulipes* (Lucas, 1857)), whereas one recovered them as a single species. One of the former two analyses also suggested partitioning within *L. colleni* Henriques, Miñano & Pérez-Zarcos, 2018 (Fig. 5C).

Considering the genetic distance of nearly 2% from *L. phoenixi*, together with the distinct differences in coloration, conductor morphology, and geographic distribution, we treat the specimen from Saudi Arabia as a separate species.

##### Distribution.

Known only from the type locality in the Eastern Province, northeastern Saudi Arabia. This is the first record of *Loureedia* Miller, Griswold, Scharff, Řezáč, Szűts & Marhabaie, 2012 from this country and the southernmost known record of the genus.

##### Etymology.

This species is named after Casey J. Melancon, an American who collected the holotype and is working as a lead avionics and aircraft electrical technician in Saudi Arabia.

###### Family Filistatidae Ausserer, 1867

#### Sahastata sp.

Taxon classificationAnimaliaAraneaeFilistatidae

819CD96D-EF55-5672-AB12-13FA164A231E

Figs 6D, 7A, 7B

Sahastata infuscata : Magalhaes et al. 2020: 242, fig. 25C (♀).

##### Material.

Saudi Arabia: Jazan Prov.: • 1♀ (ZMUT), Dayhamah, 16°31'56.8"N, 42°47'39.2"E, 3.8.2024 (I.M. Fageeh).

##### Description.

**Figure 6.**
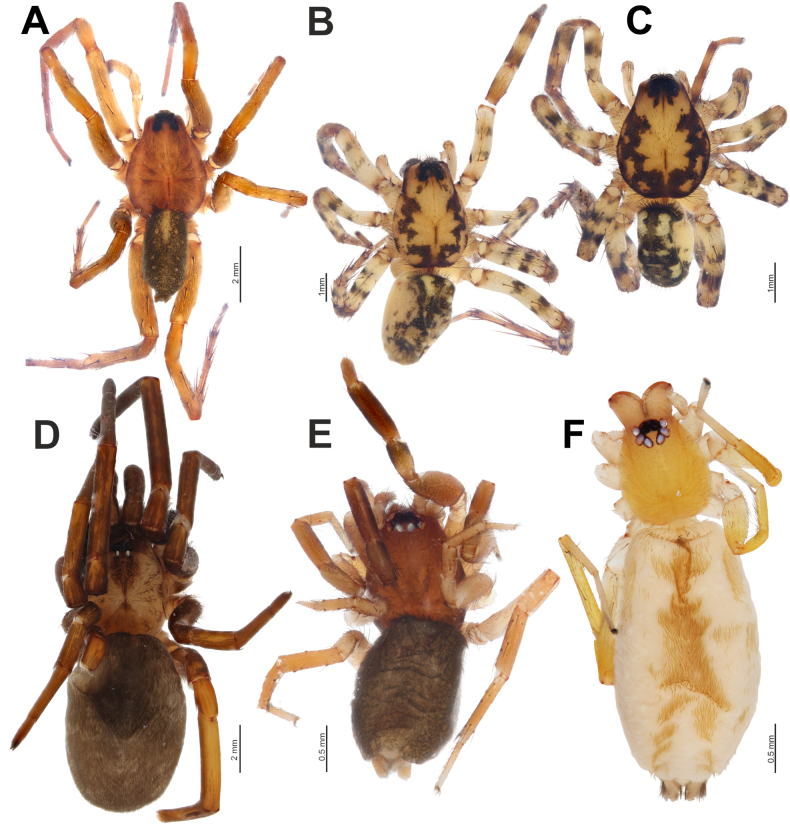
Habitus of *Trochosa dentichelis* (**A**), *Arctosa formosa* sp. nov. (**B, C**), *Sahastata* sp. (**D**), *Synaphosus dulcicola* sp. nov. (**E**), and *Prodidomus emiratus* sp. nov. (**F**), dorsal view. **A, B**. Males; **C–F**. Females.

**Female**. Habitus as in Fig. 6D. Total length 11.20. Carapace 4.60 long, 3.15 wide. Carapace pale brown, with dark brown clypeus and post ocular marking; chelicerae, maxillae, labium, and sternum covered with dark brown pubescens. Abdomen and spinnerets greyish brown. Legs brown. Measurements of legs: I: 17.85 (4.85, 1.80, 4.70, 4.35, 2.15), II: 12.95 (3.65, 1.55, 3.10, 2.95, 1.70), III: 10.45 (3.05, 1.55, 2.25, 2.40, 1.20), IV: 13.30+missing tarsus (4.20, 1.80, 3.85, 3.45, missing).

**Figure 7.**
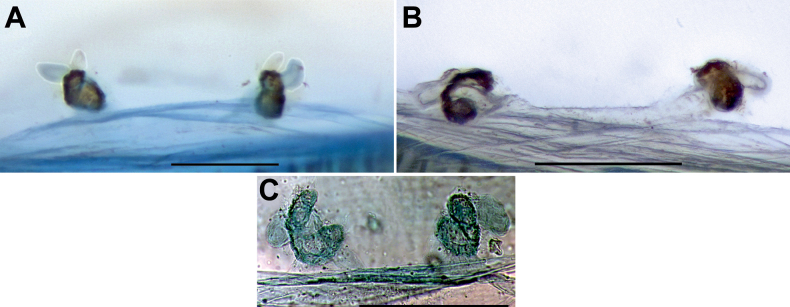
Endogyne of *Sahastata* sp. from Saudi Arabia (**A, B**) and *S. infuscata* (**C**), dorsal view. **A, B**. Spermathecae in different positions. **C**. Reproduced from Marusik et al. (2017). Scale bars: 0.2 mm.

Endogyne as in Fig. 7A, B; spermathecae well separated, with elongate digitiform membranous part, surrounded near mid-region by reniform glandular part.

**Male**. Likely unknown (see below).

##### Remarks.

This specimen has an endogyne identical to that of *S. aravaensis* Ganem, Magalhaes, Zonstein & Gavish-Regev, 2022 from the Southern Levant (cf. Fig. 7A vs Gavish-Regev et al. 2022: figs 9A, B, 10G). However, it differs in overall coloration (brown with a distinct cephalic marking vs much darker brown to black with only faint contrast between the cephalic and thoracic regions; cf. Fig. 6D vs Gavish-Regev et al. 2022: figs 11B, D, 12C). We therefore refrain from assigning the present material to any species or from treating it as a new one until conspecific males from both this population and *S. aravaensis* become available. This represents the first record of the family Filistatidae from Saudi Arabia.

A single female specimen from northeastern Yemen, tentatively identified as *S. infuscata* (Kulczyński, 1901) by Magalhaes et al. (2020), has an endogyne nearly identical to that of our specimen from Saudi Arabia (cf. Fig. 7A vs Magalhaes et al. 2020: fig. 25C), but rather different from that of the holotype of *S. infuscata* (cf. Fig. 7A vs Fig. 7C). Given this similarity and the close proximity of the two collection sites (aerial distance ~100 km), we consider the two specimens to be conspecific, but not attributable to *S. infuscata*. Since their endogyne morphology is identical to that of *S. aravaensis*, the diagnostic characters provided for *S. aravaensis* in Gavish-Regev et al. (2022: 18) are also applicable to distinguish these specimens from *S. infuscata*.

###### Family Gnaphosidae Banks, 1892

#### Berlandina plumalis

Taxon classificationAnimaliaAraneaeGnaphosidae

(O. Pickard-Cambridge, 1872)

252AE2CC-E946-5B59-8F33-B71E5E1F94B8

##### Material.

UAE: Ras Al Khaimah: • 1♂ (DWLC), W. Daftah, 25°16'51.6"N, 56°11'13.2"E, 444 m, 14.8.2025 (M.R. Sharaf).

##### Distribution.

Mediterranean to Central Asia and Afghanistan, Myanmar. New genus record for the UAE.

#### Drassyllus crimeaensis

Taxon classificationAnimaliaAraneaeGnaphosidae

Kovblyuk, 2003

7E2D46A4-6382-52D1-B12A-1F1C6974BED8

##### Material.

Lebanon: Keserwan-Jbeil Gov.: • 1♂ (ZMUT), Faitroun, 33°59'32"N, 35°44'13"E, 1200 m, 21.4.2022 (C. Reuter).

##### Distribution.

North Macedonia and Crimea (Ukraine) to Iran. New genus record for Lebanon, with the present material representing the southernmost known record of the species.

#### Leptopilos hajarensis

Taxon classificationAnimaliaAraneaeGnaphosidae

Zamani & Marusik, sp. nov.

7020B029-937E-5D6D-A834-A22A706030F3

https://zoobank.org/197DDDDC-203D-4C09-B8D9-9627ECD57633

Fig. 8A–G

##### Type material.

***Holotype*** • ♀ (DWLC), UAE: Sharjah: W. Shees, 25°17'24.0"N, 56°14'42.0"E, 306 m, 27.8.2025 (M.R. Sharaf).

##### Diagnosis.

**Figure 8.**
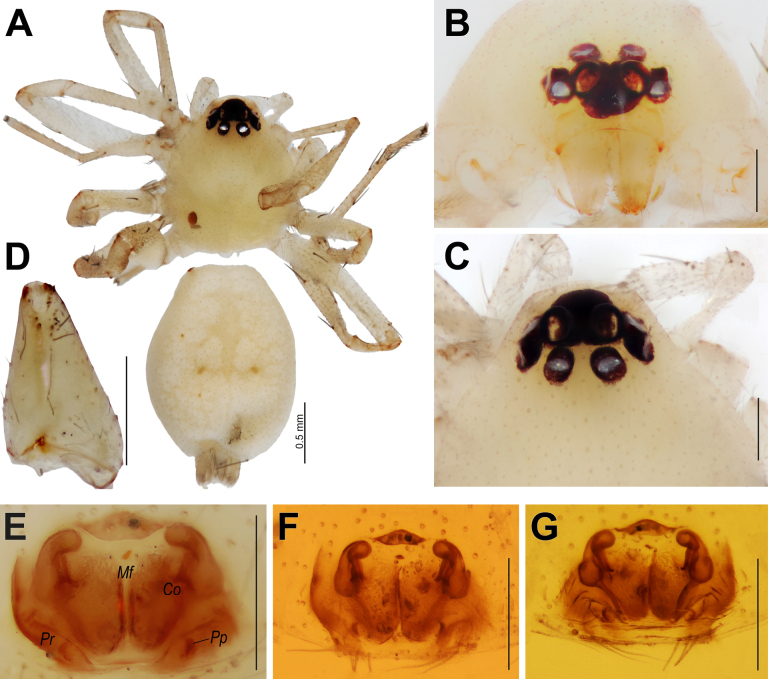
*Leptopilos hajarensis* sp. nov., female. **A**. Habitus, dorsal view; **B**. Cephalothorax, frontal view; **C**. Eyes, dorsal view; **D**. Chelicera, prolateral view; **E**. Intact epigyne, ventral view; **F, G**. Macerated epigyne, ventral and dorsal views. Abbreviations: *Co* = copulatory opening; *Mf* = median furrow; *Pp* = posterior pocket; *Pr* = posterior ridge. Scale bars: 0.2 mm, unless stated otherwise.

The new species is similar to *L. levantinus* Levy, 2009, but differs in having a distinct median furrow (*Mf*) clearly visible on both the epigyne and endogyne, and in the anterior part of the receptacles being smaller than the posterior part (vs anterior part globular and wider than the posterior part) (cf. Fig. 8E–G vs Chatzaki et al. 2002: figs 74, 75).

##### Description.

**Female**. Habitus as in Fig. 8A. Total length 2.90. Carapace 1.30 long, 1.05 wide. Body and appendages almost completely pale beige. Chelicera as in Fig. 8D, with two pro- and three retromarginal teeth. Measurements of legs: I: 4.00 (1.10, 0.45, 0.95, 0.85, 0.65), II: 4.05 (1.10, 0.45, 1.00, 0.85, 0.65), III: 2.60 (0.75, 0.35, 0.55, 0.55, 0.40), IV: 3.25 (1.40, 0.45, 1.05, 1.35, 0.35).

Epigyne as in Fig. 8E–G; plate ~1.5× wider than long, with distinct anterior hood and pair of small posterior pockets (*Pp*); fovea ~1.4× longer than wide, posterior part with oblique ridges (*Pr*); copulatory openings (*Co*) located at anterior third of fovea; copulatory ducts straight, more strongly sclerotized than other parts of endogyne; receptacles longitudinal, posterior part wider than anterior.

**Male**. Unknown.

##### Distribution.

Known only from the type locality in Sharjah, northern UAE. This is the first record of the genus *Leptopilos* Levy, 2009 from this country.

##### Etymology.

The specific epithet refers to the Hajar Mountains, the type locality of the new species.

#### Minosiella intermedia

Taxon classificationAnimaliaAraneaeGnaphosidae

Denis, 1958

68D2E91D-8E63-5A66-84D8-A1C148E4CF63

##### Material.

UAE: Ras Al Khaimah: • 1♀ (DWLC), Kedra, 25°13'12.0"N, 56°01'40.8"E, 248 m, 31.7.2025 (M.R. Sharaf); Sharjah: • 5♂1♀ (DWLC), W. Shees, 25°17'24.0"N, 56°14'42.0"E, 418 m, 14.8.2025 (M.R. Sharaf); • 1♂ (DWLC), Kalba, W. Al-Helo, 24°56'34.8"N, 56°12'03.6"E, 256 m, 20.8.2025 (M.R. Sharaf).

##### Distribution.

Egypt to Central Asia and Afghanistan. New genus record for the UAE.

#### Synaphosus dulcicola

Taxon classificationAnimaliaAraneaeGnaphosidae

Zamani & Marusik, sp. nov.

EEBE7370-AB4B-5E8E-88AB-D7581C1BCC00

https://zoobank.org/396C449A-553F-41AD-BD3B-0D378417E7F0

Figs 6E, 9B–D

##### Type material.

***Holotype*** • ♀ (DWLC), UAE: Sharjah: Kalba, W. Al-Helo, 24°56'34.8"N, 56°12'03.6"E, 265 m, 20.8.2025 (M.R. Sharaf).

##### Comparative material.

**Figure 9.**
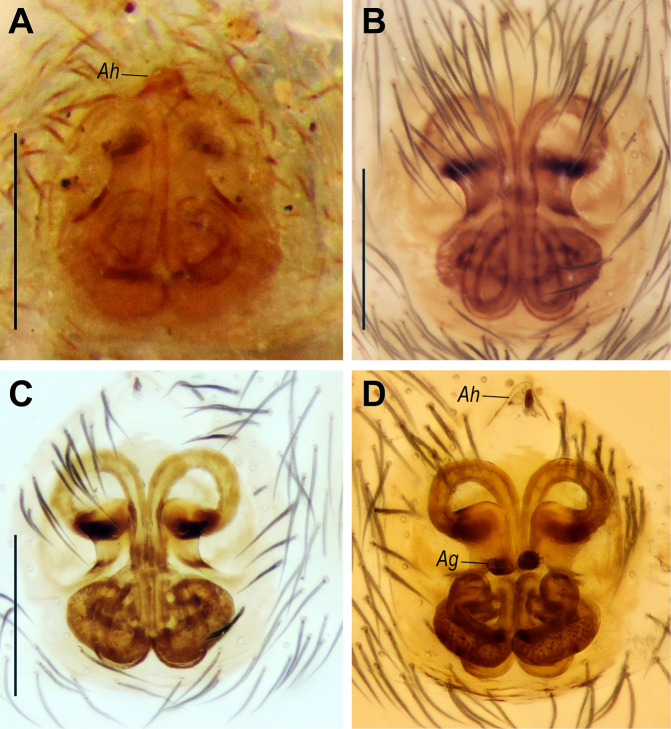
Epigyne of *Synaphosus gracillimus* (**A**) and *S. dulcicola* sp. nov. (**B–D**). **A, B**. Intact, ventral view; **C, D**. Macerated, ventral and dorsal views. Abbreviations: *Ag* = accessory gland; *Ah* = anterior hood. Scale bars: 0.2 mm.

*Synaphosus gracillimus* (O. Pickard-Cambridge, 1872) (Fig. 9A): Israel: Southern Dist.: • 1♀ (SMNHTAU), NW Negev Desert, Nahal Besor, 31°08'N, 34°37'E, 200 m, 8.4.2010 (L. Friedman & C. Dress).

##### Diagnosis.

The new species is similar to *S. gracillimus*, known from Egypt and Israel, but differs in having the anterior hood (*Ah*) located anterior to the anterior loops of the copulatory ducts (vs at the same level), the anterior loop almost round (vs oval), relatively larger accessory glands (*Ag*), and anterior loops spanning wider than the receptacles (vs the opposite) (cf. Fig. 9B–D vs Fig. 9A and Ovtsharenko et al. 1994: figs 56, 57).

##### Description.

**Female**. Habitus as in Fig. 6E. Total length 2.80. Carapace 1.20 long, 1.05 wide. Carapace and chelicerae brown; sternum, labium, and maxillae pale brown. Legs pale brown; patellae and tibiae I and II almost entirely dark brown. Abdomen dark grey dorsally, beige ventrally. Spinnerets pale brown. Measurements of legs: I: 3.15 (0.95, 0.55, 0.70, 0.50, 0.45), II: 3.00 (0.90, 0.45, 0.70, 0.50, 0.45), III: 2.45 (0.70, 0.40, 0.45, 0.50, 0.40), IV: 3.70 (0.95, 0.60, 0.80, 0.80, 0.55).

Epigyne as in Fig. 9B–D; plate ~1.3× longer than wide; anterior hood (*Ah*) located anterior to anterior loops of copulatory ducts, separated by ~2× its length; fovea absent; copulatory ducts situated in median part of plate, directed laterally; ducts long, forming anterior and posterior loops; anterior loops almost round, posterior loops elongate-oval; copulatory ducts with globular accessory glands (*Ag*) lacking stalks; receptacles looped, span of anterior loops wider than that of receptacles.

**Male**. Unknown.

##### Distribution.

Known only from the type locality in Sharjah, northern UAE. This is the first record of the genus *Synaphosus* Platnick & Shadab, 1980 from this country.

##### Etymology.

The specific epithet is a compound of the Latin word *dulcis*, meaning sweet, and the Latin suffix -*cola*, meaning inhabitant or dweller. It refers to the type locality of the species, situated in Wadi Al-Helo (“Sweet Wadi”).

#### Trichothyse golan

Taxon classificationAnimaliaAraneaeGnaphosidae

(Levy, 1999)

015C6906-12CB-5F0F-8BE1-66FCFFC8147B

Fig. 10A–D

Poecilochroa golan Levy, 1999: 435, figs 15, 16 (♀).
Trichothyse golan : Sankaran et al. 2025: 59.

##### Material.

Lebanon: Mount Lebanon Gov.: • 1♂ (ZMUT), Sahih Valley, 33°54'N, 35°36'E, 14.8.2022 (A. Martinez); Golan Heights: • 1♀ (SMNHTAU), Mt. Hermon Area, Panyas, 33°15'N, 35°42'E, 380 m, 17.6.2020 (S. Zonstein); • 2♀ (SMNHTAU), Mt. Hermon Area, Senir, 33°14'N, 35°40'E, 250 m, 24.5.2012 (S. Zonstein); West Bank: • 1♀ (SMNHTAU), Judean Hills, Nahshon Jnc., 31°49'N, 34°55'E, 130 m, 6–23.5.2020 (W. Kuslitzky); • 3♂ (SMNHTAU), same locality, 23.5.–3.6.2020 (W. Kuslitzky); • 2♀ (SMNHTAU), same locality, 14–24.6.2020 (W. Kuslitzky); • 1♀ (SMNHTAU), same locality, 1–12.7.2020 (W. Kuslitzky); • 1♀ (SMNHTAU), same locality, 13–30.6.2022 (W. Kuslitzky).

##### Diagnosis.

**Figure 10.**
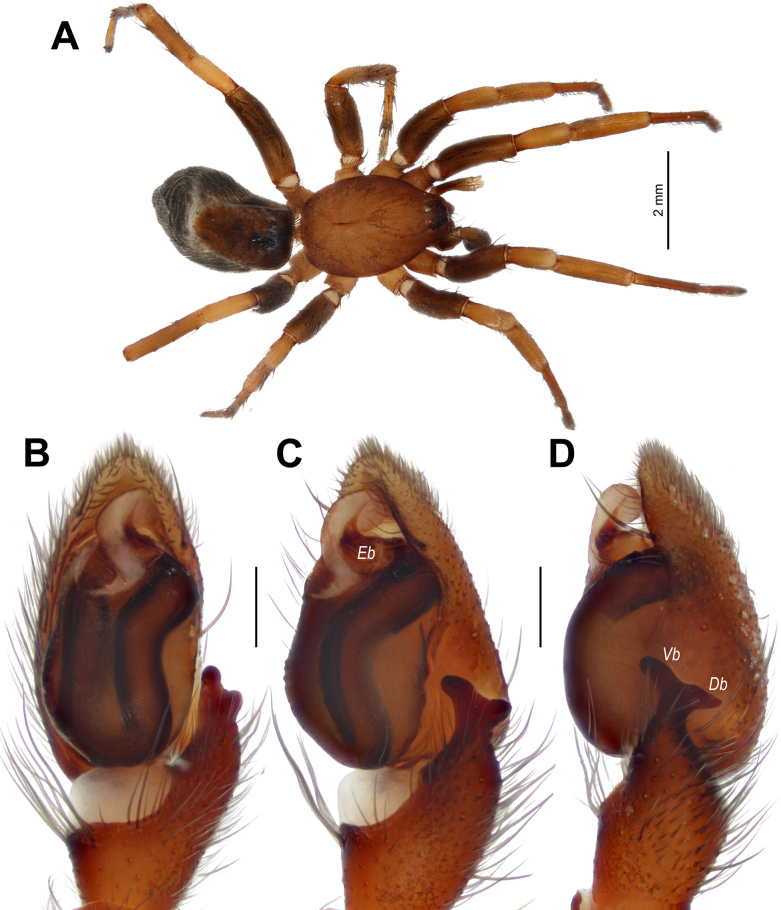
*Trichothyse golan*, male. **A**. Habitus, dorsal view; **B–D**. Palp, ventral, retroventral, and retrolateral views. Abbreviations: *Db* = dorsal branch of the tibial apophysis; *Eb* = base of the embolus; *Vb* = ventral branch of the tibial apophysis. Scale bars: 0.2 mm, unless stated otherwise.

The male of *T. golan* is most similar to that of *T. loricata* (Kritscher, 1996), known from Sicily, in having a tibial apophysis with anterior branches almost subequal in length and forming an almost straight line, but differs in having the dorsal branch (*Db*) rounded apically and the ventral branch (*Vb*) pointed (vs the opposite). The two species also differ in the shape of the embolus: *T. golan* has a wide embolic base (*Eb*) and a claw-like tip, whereas in *T. loricata* the base is of uniform width and the embolus is straight (cf. Fig. 10B–D vs Bauer 2022: fig. 1c–e).

##### Description.

**Male** (specimen from Lebanon). Habitus as in Fig. 10A. Total length 6.10. Carapace 2.75 long, 2.05 wide. Carapace and chelicerae pale brown; sternum, maxillae, and labium yellowish brown. Femora dark brown, other segments pale brown. Abdomen dark grey, paler ventrally, dorsally with large brownish scutum covering almost half of its surface. Spinnerets dark grey, paler apically. Measurements of legs: I: 6.80 (2.00, 1.20, 1.45, 1.30, 0.85); II: 6.65 (1.95, 1.15, 1.40, 1.30, 0.85); III: 5.95 (1.85, 0.95, 1.15, 1.25, 0.75); IV: 7.85 (2.25, 1.15, 1.65, 2.05, 0.75).

Palp as in Fig. 10B–D; retrolateral tibial apophysis with two branches of subequal length, forming an almost straight line; ventral branch (*Vb*) with rounded tip, dorsal branch (*Db*) pointed; cymbium ~2× longer than wide, with two anterior spines on each side; bulb ~1.4× longer than wide; spermophor wide, forming a single loop, prolateral part straight, retrolateral part slightly bent; conductor wide, its tip as wide as embolic base in retrolateral view; embolus with wide base (*Eb*), forming a right angle with the base.

**Female**. See Levy (1999).

##### Remarks.

This species was previously known from the female only.

##### Distribution.

Previously known only from the type locality in the Golan Heights. This is the first record of the genus *Trichothyse* Tucker, 1923 from Lebanon, and the northernmost known record of the species.

#### Zelotes laetus

Taxon classificationAnimaliaAraneaeGnaphosidae

(O. Pickard-Cambridge, 1872)

15EA6BCE-909D-5B1C-B5B6-485ED7AEA783

##### Material.

Kuwait: Al Ahmadi Gov.: • 1♀ (ZMUT), Sabah Al Ahmad Vill., 28°46'N, 48°03'E 4.6.2022 (A. Martinez); UAE: Sharjah: • 1♂ (DWLC), W. Shees, 25°17'24.0"N, 56°14'42.0"E, 418 m, 14.8.2025 (M.R. Sharaf).

##### Distribution.

North Africa to Senegal and Kenya, Portugal, France, Greece (Crete) to the Middle East; introduced to Hawaii, USA, Mexico, Peru, Galapagos, and Ascension. New genus records for Kuwait and the UAE.

###### Family Linyphiidae Blackwall, 1859

#### Nesioneta arabica

Taxon classificationAnimaliaAraneaeLinyphiidae

Tanasevitch, 2010

0C0F2228-5999-5750-8ED9-FD872D19F731

##### Material.

UAE: Ras Al Khaimah: • 1♂ (DWLC), W. Daftah, 25°16'51.6"N, 56°11'13.2"E, 444 m, 14.8.2025 (M.R. Sharaf); Sharjah: • 7♂1♀ (ZMUT), W. Shees, 25°17'24.0"N, 56°14'42.0"E, 418 m, 14.8.2025 (M.R. Sharaf).

##### Distribution.

Endemic to the UAE and known from all emirates except Ajman and Umm Al-Quwain (Tanasevitch 2010).

###### Family Lycosidae Sundevall, 1833

#### Arctosa

Taxon classificationAnimaliaAraneaeLycosidae

C.L. Koch, 1847

F5A7AB64-B3D3-57F2-864E-3760E2409415

##### Remarks.

This is one of the largest genera in the family, with 167 valid species (WSC 2026). Ten genera are currently regarded as junior synonyms of *Arctosa*. The genus has an almost global distribution, being absent only from Australasia, and has long served as a “wastebasket” for species of uncertain placement. More than one third of the species are known from a single sex only: 54 from females, nine from males, and three from juveniles (WSC 2026). Six species have never been illustrated, and most species from Africa and Central Asia are poorly illustrated. Species of this genus have never been formally grouped; consequently, it is difficult to assess which species are closely related to the new species described below.

#### Arctosa formosa

Taxon classificationAnimaliaAraneaeLycosidae

Zamani & Marusik, sp. nov.

C77AEF2B-F43C-593C-AB54-68E4E69AD8AA

https://zoobank.org/FBA96130-45D7-4BC3-B202-CA5DD26E10B5

**Figure 11.**
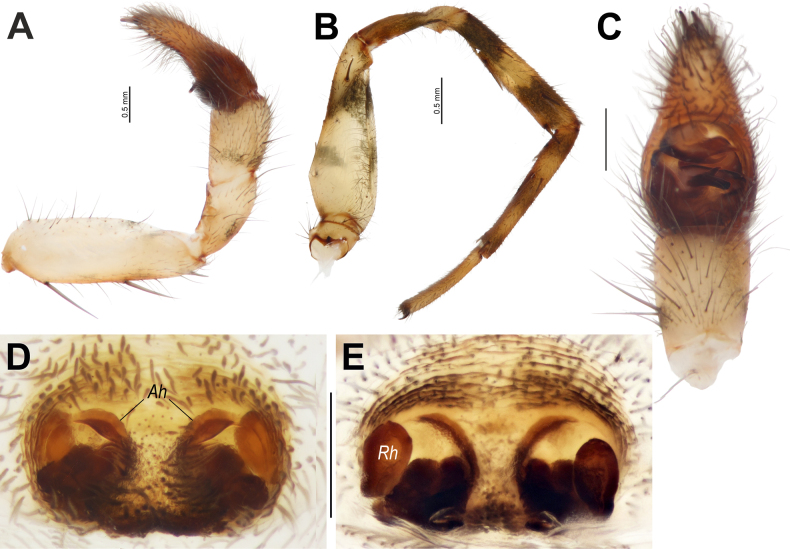
*Arctosa formosa* sp. nov., male (**A–C**) and female (**D, E**). **A**. Whole palp, retrolateral view; **B**. Leg I, prolateral view; **C**. Palp, ventral view; **D, E**. Macerated epigyne, ventral and dorsal views. Abbreviations: *Ah* = anterior hoods; *Rh* = head of the receptacle. Scale bars: 0.2 mm, unless stated otherwise.

**Figure 12.**
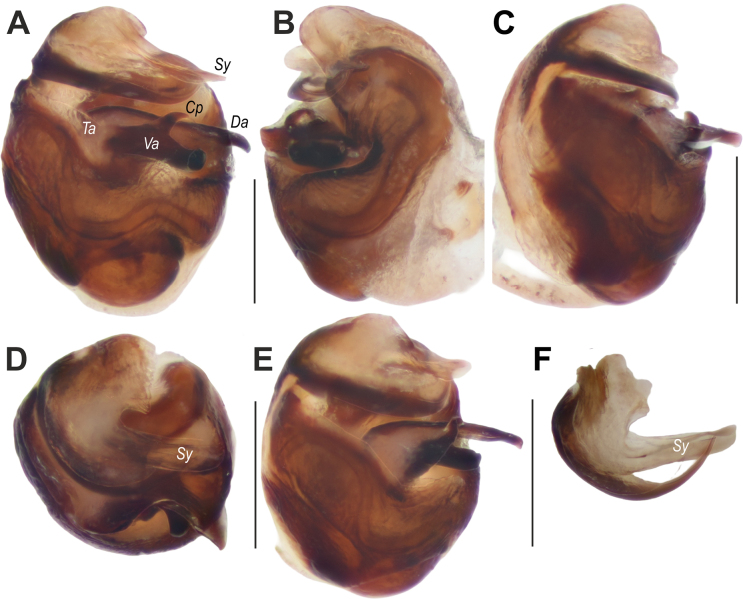
*Arctosa formosa* sp. nov., bulb. **A**. Ventral view; **B**. Retrolateral view; **C**. Prolateral view; **D**. Distal view; **E**. Distoproventral view; **F**. Embolic division, distal view. Abbreviations: *Cp* = claw-like process; *Da* = dorsal arm of the tegular apophysis; *Sy* = synembolus; *Ta* = tegular apophysis; *Va* = ventral arm of the tegular apophysis. Scale bars: 0.2 mm.

Figs 6B, 6C, 11A–E, 12A–F

##### Type material.

***Holotype*** • ♂ (DWLC), UAE: Sharjah: W. Shees, 25°17'24.0"N, 56°14'42.0"E, 324 m, 28.8.2025 (M.R. Sharaf). ***Paratypes*** • 1♂1♀ (ZMUT), collected together with the holotype.

##### Diagnosis.

The new species differs from West Palaearctic *Arctosa* by its variegated pattern, otherwise known only in *Arctosa* s.l. from Southeast Asia (e.g., *A. gougu* Chen & Song, 1999; *A. vaginalis* Yu & Song, 1988; *A. zhaojingzhaoi* Li, 2016; see Wang et al. 2012 and Pan et al. 2016). The epigyne of *A. formosa* sp. nov. is somewhat similar to that of *A. vaginalis* from South China, with the septum margins nearly parallel and slightly widening posteriorly, but differs in relative length (length/width ratio 1.0 vs 1.5) and in having receptacles lacking stalks (vs stalk present) (cf. Fig. 11D, E vs Wang et al. 2012: fig. 8C, D). The male of *A. formosa* sp. nov. differs from congeners in possessing a claw-like process (*Cp*) on the ventral arm (*Va*) of the tegular apophysis (*Ta*; Fig. 12A), absent in other congeners.

##### Description.

**Male** (holotype). Habitus as in Fig. 6B. Total length 5.40. Carapace 3.00 long, 2.35 wide. Carapace yellowish brown, with dark brown wave-shaped lateral bands and post ocular small patches; chelicerae and labium reddish brown; sternum and maxillae yellowish brown. Legs pale yellowish brown, darker distally, with numerous annulations and broad markings (Fig. 11B). Abdomen dark grey dorsally, with numerous pale and grey markings medially and submedially, pale yellowish brown ventrally. Spinnerets pale yellowish brown. Measurements of palp and legs: palp: 2.90 (1.05, 0.55, 0.45, -, 0.85), I: 7.65 (2.20, 1.05, 1.70, 1.70, 1.00), II: 6.45 (1.70, 1.05, 1.30, 1.50, 0.90), III: 6.40 (1.75, 0.95, 1.15, 1.60, 0.95), IV: 9.85 (2.65, 1.30, 2.05, 2.70, 1.15).

Palp as in Figs 11A, 11C, 12A–F; femur somewhat swollen, ~3× longer than wide; patella + tibia as long as femur; tibia swollen, almost as wide as cymbium; cymbium 1.25× wider than tibia (ventral view), 1.8× longer than wide, with two claws on tip, tip ~0.66× cymbial length; bulb 1.4× longer than wide; spermophor thin in posterior and prolateral parts of tegulum, roundly bent retrolaterally, ventrally and prolaterally; tegular apophysis (*Ta*) with two arms: dorsal (*Da*) straight, pointed; ventral (*Va*) bifurcated, with anterior claw-like process (*Cp*) and hooked tip; conductor (tegular part) indistinct; embolic division with long, straight, broad synembolus (*Sy*); embolus roundly bent, tapering from base to tip.

**Female**. Habitus as in Fig. 6C. Total length 7.15. Carapace 3.60 long, 2.60 wide. Coloration as in male. Measurements of legs: I: 8.85 (2.45, 1.45, 1.90, 1.90, 1.15), II: 7.60 (2.00, 1.30, 1.60, 1.65, 1.05), III: 7.30 (2.10, 1.10, 1.35, 1.85, 0.90), IV: 11.30 (3.05, 1.55, 2.40, 3.10, 1.20).

Epigyne as in Fig. 11D, E; plate 1.6× wider than long; septum short, broad, as long as wide, base unmodified, entirely covered with setae, anteriorly with hood-like structures (*Ah*); heads of receptacles (*Rh*) ~1.5× longer than wide, lacking stalks (ventral and dorsal views), separated by >3× their width, not extending beyond anterior margin of septum.

##### Remarks.

In their survey of the lycosids of the UAE, Alderweireldt and Jocqué (2017) reported two apparently undescribed species of *Arctosa*, which they neither illustrated nor formally described due to the absence of a comprehensive regional revision. One of these may correspond to the new species described here.

##### Distribution.

Known only from the type locality in Sharjah, northern UAE.

##### Etymology.

The specific epithet is a Latin adjective meaning “beautiful.”

#### Hogna effera

Taxon classificationAnimaliaAraneaeLycosidae

(O. Pickard-Cambridge, 1872)

209721AB-8330-51DD-A693-B8CE3FA002D7

##### Material.

UAE: Ras Al Khaimah: • 1♂ (DWLC), W. Daftah, 25°16'51.6"N, 56°11'13.2"E, 431 m, 14.8.2025 (M.R. Sharaf).

##### Distribution.

Middle East. In the UAE, it was previously reported from Fujairah, Ras Al Khaimah (Alderweireldt and Jocqué 2017), and Abu Dhabi (Logunov 2020).

#### Trochosa dentichelis

Taxon classificationAnimaliaAraneaeLycosidae

Buchar, 1997

32B3492E-67BD-598C-AA82-FD4CD19588FC

**Figure 13.**
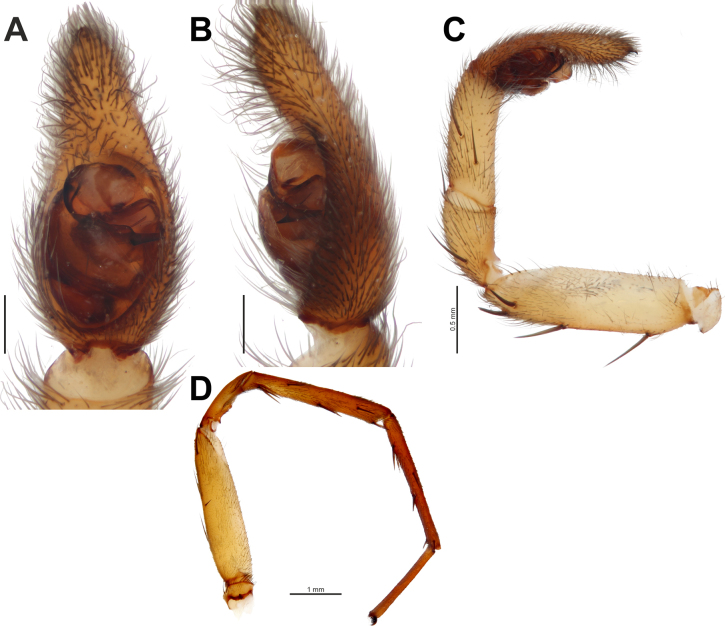
*Trochosa dentichelis*, male. **A, B**. Palp, ventral and retrolateral views; **C**. Whole palp, prolateral view; **D**. Leg I, prolateral view. Scale bars: 0.2 mm, unless stated otherwise.

**Figure 14.**
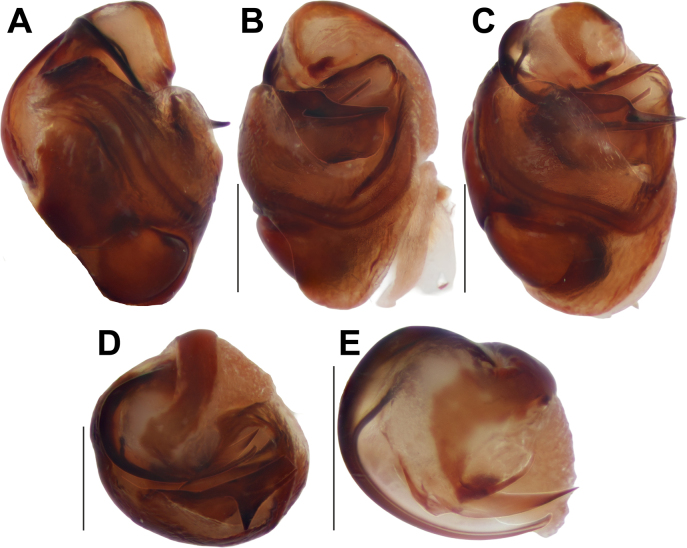
*Trochosa dentichelis*, bulb. **A**. Prolateral view; **B**. Retrolateral view; **C**. Ventral view; **D**. Distal view; **E**. Embolic division, distal view. Scale bars: 0.2 mm.

Figs 6A, 13A–D, 14A–E

##### Material.

UAE: Sharjah: • 6♂ (DWLC, ZMUT), W. Shees, 25°17'24.0"N, 56°14'42.0"E, 418 m, 14.8.2025 (M.R. Sharaf).

##### Distribution.

Previously known from northern India to Bhutan. New genus record for the UAE, with the present material representing the westernmost known record of the species.

#### Wadicosa fidelis

Taxon classificationAnimaliaAraneaeLycosidae

(O. Pickard-Cambridge, 1872)

C1B860DE-5213-5E5B-ADD5-3CFEFF291729

##### Material.

UAE: Ras Al Khaimah: • 1♂1♀ (DWLC), W. Daftah, 25°16'51.6"N, 56°11'13.2"E, 444 m, 14.8.2025 (M.R. Sharaf); Sharjah: • 1♂ (DWLC), W. Shees, 25°17'24.0"N, 56°15'14.4"E, 418 m, 10.7.2025 (M.R. Sharaf).

##### Distribution.

Trans-Palaearctic and Southeast Asia: Morocco and Portugal to the Philippines and Indonesia (Sumatra). In the UAE, it was previously reported from all emirates except Ajman and Umm Al-Quwain (Feulner and Roobas 2015; Alderweireldt and Jocqué 2017).

###### Family Oecobiidae Blackwall, 1862

#### Oecobius marathaus

Taxon classificationAnimaliaAraneaeOecobiidae

Tikader, 1962

BC0150A7-8F2B-5282-9551-4113E86D7823

##### Material.

UAE: Sharjah: • 9♂7♀ (DWLC), W. Shees, 25°17'24.0"N, 56°14'42.0"E, 418 m, 14.8.2025 (M.R. Sharaf).

##### Distribution.

Tropical Africa; introduced to Brazil, Saint Helena, Ascension, India, Laos, Taiwan, Japan, and Australia (Queensland). New record for the UAE.

#### Oecobius putus

Taxon classificationAnimaliaAraneaeOecobiidae

O. Pickard-Cambridge, 1876

27E28E48-E9C2-53C5-A00D-3F3F49941065

##### Material.

Kuwait: Al Ahmadi Gov.: • 1♂ (ZMUT), Sabah Al Ahmad Vill., 28°46'N, 48°03'E 4.6.2022 (A. Martinez); UAE: Ras Al Khaimah: • 1♂ (DWLC), W. Daftah, 25°16'51.6"N, 56°11'13.2"E, 444 m, 14.8.2025 (M.R. Sharaf); Sharjah: • 1♂ (DWLC), W. Shees, 25°17'24.0"N, 56°15'14.4"E, 418 m, 10.7.2025 (M.R. Sharaf).

##### Distribution.

South Africa, Egypt, Cyprus, Sudan to Azerbaijan, Afghanistan, and India; introduced to the USA and Mexico. New records for Kuwait and the UAE.

###### Family Philodromidae Thorell, 1869

#### Thanatus imbecillus

Taxon classificationAnimaliaAraneaePhilodromidae

L. Koch, 1878

83BF6968-0640-58FE-A643-7522ECB7B951

##### Material.

Lebanon: Keserwan-Jbeil Gov.: • 1♂ (ZMUT), Faitroun, 33°59'32"N, 35°44'13"E, 1200 m, 21.4.2022 (C. Reuter).

##### Distribution.

Greece to Central Asia. New record for Lebanon.

#### Thanatus setiger

Taxon classificationAnimaliaAraneaePhilodromidae

(O. Pickard-Cambridge, 1872)

82D7058A-7C3D-50F9-B13C-D957A30F1BD4

##### Material.

Lebanon: Keserwan-Jbeil Gov.: • 1♂ (ZMUT), Faitroun, 33°59'32"N, 35°44'13"E, 1200 m, 21.4.2022 (C. Reuter).

##### Distribution.

Morocco to Iran and the UAE. New record for Lebanon.

###### Family Prodidomidae Simon, 1884

#### Prodidomus

Taxon classificationAnimaliaAraneaeProdidomidae

Hentz, 1847

6AE07405-9943-5AB8-AB18-A5FA79A105A3

##### Remarks.

This is a large genus with 54 named species distributed worldwide (WSC 2026). It has never been properly revised, and many species lack illustrations of the endogyne. Based on published figures of the epigynes and palps, the genus is likely polyphyletic. Several species from the Mediterranean, Arabian Peninsula, and Sudan have similar epigynes and may represent junior synonyms of the cosmopolitan *P. rufus* Hentz, 1847: *P. aurantiacus* Simon, 1890 (Yemen), *P. bicolor* Denis, 1957 (Sudan), *P. geniculosus* Dalmas, 1919 (Tunisia), and *P. rollasoni* Cooke, 1964 (Libya).

#### Prodidomus emiratus

Taxon classificationAnimaliaAraneaeProdidomidae

Zamani & Marusik, sp. nov.

4C399C5F-F895-520D-BEB0-E72C961BF439

https://zoobank.org/AB22D7E3-8EBE-40D9-8288-C3769F2B1F41

Figs 6F, 15A–C

##### Type material.

***Holotype*** • ♀ (DWLC), UAE: Ras Al Khaimah: Masafi, 25°14'16.8"N, 56°09'54.0"E, 430 m, 29.10.2025 (M.R. Sharaf).

##### Diagnosis.

**Figure 15.**
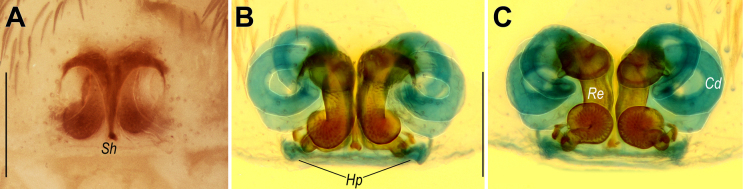
Epigyne of *Prodidomus emiratus* sp. nov. **A**. Intact, ventral view; **B, C**. Macerated, ventral and dorsal views. Abbreviations: *Cd* = copulatory duct; *Hp* = hoods of the posterior plate; *Re* = receptacle; *Sh* = posterior septal hood. Scale bars: 0.2 mm.

The new species is most similar to *P. nigellus* Simon, 1890, known from Yemen. Both species have a thin posterior plate, but differ in the size of the septal hood (*Sh*) and the sclerotization of the septal base: the new species has a small, pit-like hood and an unsclerotized base, whereas *P. nigellus* has a large hood and a base as wide as the fovea, well sclerotized (cf. Fig. 15A–C vs Dalmas 1919: fig. 28). The endogyne of *P. nigellus* has never been illustrated and cannot be compared.

##### Description.

**Female**. Habitus as in Fig. 6F. Total length 3.55. Carapace 1.10 long, 0.85 wide. Carapace and legs yellow; chelicerae beige; sternum, labium, maxillae, abdomen, and spinnerets pale beige. Measurements of legs: I: missing, II: 2.90 (0.75, 0.50, 0.70, 0.55, 0.40), III: 2.55 (0.70, 0.40, 0.55, 0.55, 0.35), IV: 4.00 (1.10, 0.55, 1.00, 0.80, 0.55).

Epigyne as in Fig. 15A–C; plate 1.2× wider than long; fovea divided by tapering septum, septum with pair of anterior hoods and small posterior hood (*Sh*); posterior part of fovea weakly sclerotized; thin transverse posterior plate posterior to fovea, almost indistinct on non-dissected epigyne, with pair of hoods (*Hp*); copulatory ducts (*Cd*) long, wide, forming three circular loops; receptacles (*Re*) tubular, anterior portion straight and parallel, posterior portion roundly bent.

**Male**. Unknown.

##### Distribution.

Known only from the type locality in Ras Al Khaimah, northern UAE.

##### Etymology.

The specific epithet refers to the distribution of the species in the UAE.

###### Family Salticidae Blackwall, 1841

#### Cyrba ocellata

Taxon classificationAnimaliaAraneaeSalticidae

(Kroneberg, 1875)

2C4323F7-2DA1-5228-87F6-46F572D39EA8

##### Material.

UAE: Sharjah: • 1♀ (DWLC), W. Shees, 25°17'24.0"N, 56°15'14.4"E, 418 m, 28.8.2025 (M.R. Sharaf).

##### Distribution.

Eastern Africa to India and Indonesia, Caucasus to Central Asia and China; introduced to Australia (Queensland). In the UAE, it was previously reported from Ras Al Khaimah (Wesołowska and van Harten 2010, 2011); new record for Sharjah.

#### Langona pallida

Taxon classificationAnimaliaAraneaeSalticidae

Prószyński, 1993

D5C08189-009C-5C64-B3B3-D0299F128D66

##### Material.

UAE: Sharjah: • 7♂5♀ (DWLC), W. Shees, 25°17'24.0"N, 56°14'42.0"E, 418 m, 14.8.2025 (M.R. Sharaf).

##### Distribution.

Saudi Arabia, UAE, and Afghanistan. In the UAE, it has been reported from all emirates except Dubai (Wesołowska and van Harten 2010, 2011, 2020).

#### Phlegra pusilla

Taxon classificationAnimaliaAraneaeSalticidae

Wesołowska & van Harten, 1994

AD5FDBB7-B86B-5C00-AC94-F48DBA280D81

##### Material.

UAE: Sharjah: • 2♂ (DWLC), W. Shees, 25°17'24.0"N, 56°14'42.0"E, 418 m, 14.8.2025 (M.R. Sharaf).

##### Distribution.

Senegal, Nigeria, Zimbabwe to the UAE. In the UAE, it was previously reported from Ras Al Khaimah (Wesołowska and van Harten 2010); new record for Sharjah.

#### Plexippus paykulli

Taxon classificationAnimaliaAraneaeSalticidae

(Audouin, 1826)

255C3729-5D99-599B-B40D-8FEE1E2AC882

##### Material.

Lebanon: Mount Lebanon Gov.: • 1♂1♀ (ZMUT), Sahih Valley, 33°54'N, 35°36'E, 14.8.2022 (A. Martinez).

##### Distribution.

Africa; introduced to both Americas, Europe, Middle East, South Asia, Australia, and Pacific Islands. New record for Lebanon.

###### Family Theridiidae Sundevall, 1833

#### Coscinida tibialis

Taxon classificationAnimaliaAraneaeTheridiidae

Simon, 1895

7493C7CF-FCC9-5418-B810-4BF2E508F60D

##### Material.

UAE: Sharjah: • 1♂ (DWLC), W. Shees, 25°17'24.0"N, 56°15'14.4"E, 418 m, 10.7.2025 (M.R. Sharaf).

##### Distribution.

Africa, Southern Europe to Georgia and the Southern Levant, Yemen; introduced to Thailand. New genus record for the UAE.

#### Episinus israeliensis

Taxon classificationAnimaliaAraneaeTheridiidae

Levy, 1985

4036D351-B63F-5C41-B466-1211D56309E9

Fig. 16A–F

Episinus israeliensis Levy, 1985: 92, figs 1–10 (♂♀).
Episinus israeliensis : Levy 1998: 129, figs 238–247 (♂♀; figures reproduced from Levy 1985).

##### Material.

Lebanon: Keserwan-Jbeil Gov.: • 1♂ (ZMUT), Raifoun, 33°58'N, 35°41'E, 900 m, 11.2014 (C. Reuter).

##### Remarks.

**Figure 16.**
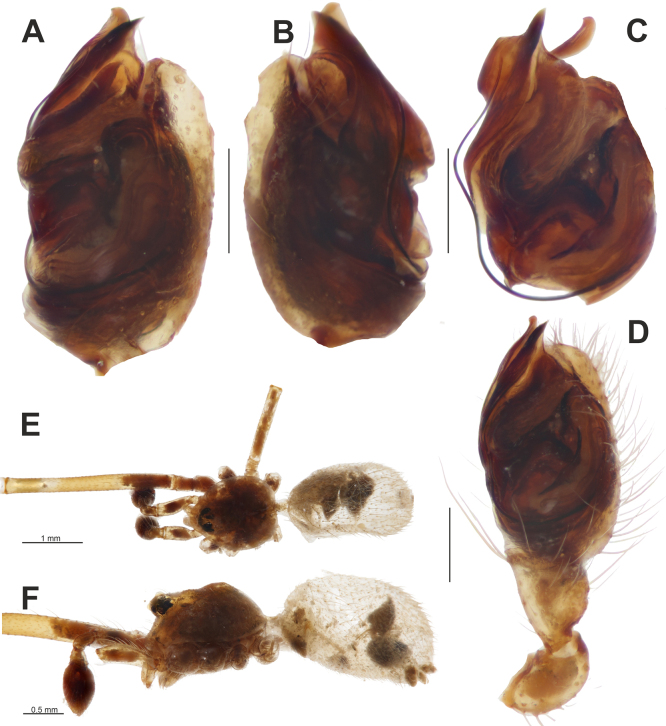
*Episinus israeliensis*, male. **A–C**. Bulb, retrolateral, prolateral, and ventral views; **D**. Palp, ventral view; **E, F**. Habitus, dorsal and lateral views. Scale bars: 0.2 mm, unless stated otherwise.

Here we provide the first photographs of the male of this species. The palpal femur is slightly swollen (Fig. 16F), a character not documented in other congeners.

##### Distribution.

Previously known only from Israel. This is the first record of the genus *Episinus* Walckenaer, 1809 from Lebanon, with the present material representing the northernmost known record of the species.

#### Latrodectus dahli

Taxon classificationAnimaliaAraneaeTheridiidae

Levi, 1959

41D2B904-8F5F-53D8-9433-B59392C3E004

##### Material.

Kuwait: Jahra Gov.: • 1♀ (SMNS-Aran-001242), nr. Camp Virginia, 29°44'N, 47°12'E, without further data; • 1♂1♀ (SMNS-Aran-001726), without further data.

##### Distribution.

Morocco, Middle East to Azerbaijan and Central Asia. New record for Kuwait.

###### Family Thomisidae Sundevall, 1833

#### Thomisus unidentatus

Taxon classificationAnimaliaAraneaeThomisidae

Dippenaar-Schoeman & van Harten, 2007

B79DE22A-5C8D-557C-A5FF-EE06DC5CC874

##### Material.

UAE: Sharjah: • 1♂ (DWLC), W. Shees, 25°16'30.0"N, 56°15'36.0"E, 306 m, 27.8.2025 (M.R. Sharaf).

##### Distribution.

Yemen, Iraq to India. New record for the UAE.

#### Thomisus zyuzini

Taxon classificationAnimaliaAraneaeThomisidae

Marusik & Logunov, 1990

B6D039A4-20DA-5921-92C5-56CD1BB04856

##### Material.

UAE: Fujairah: • 1♂ (DWLC), Al Dhaid-Masafi Rd., 25°17'42.0"N, 56°05'34.8"E, 333 m, 10.9.2025 (M.R. Sharaf).

##### Distribution.

Turkey and Cyprus to Mongolia and south to Yemen. New record for the UAE.

#### Xysticus kaznakovi

Taxon classificationAnimaliaAraneaeThomisidae

Utochkin, 1968

CF0D7FC8-6932-54B1-85C7-96DA14E7B7DA

##### Material.

Lebanon: Keserwan-Jbeil Gov.: • 1♂1♀ (ZMUT), Faitroun, 33°59'32"N, 35°44'13"E, 1200 m, 21.4.2022 (C. Reuter).

##### Distribution.

North Macedonia to Tajikistan. New record for Lebanon.

###### Family Titanoecidae Lehtinen, 1967

#### Titanoeca flavicoma

Taxon classificationAnimaliaAraneaeTitanoecidae

L. Koch, 1872

13A89A7E-B946-54C6-88C0-1653D898693D

##### Material.

Lebanon: Keserwan-Jbeil Gov.: • 3♂ (ZMUT), Faitroun, 33°59'32"N, 35°44'13"E, 1200 m, 21.4.2022 (C. Reuter).

##### Distribution.

France (Corsica) to Turkey and the Levant. New genus record for Lebanon.

###### Family Zodariidae Thorell, 1881

#### Zodarion barbarae

Taxon classificationAnimaliaAraneaeZodariidae

Bosmans, 2009

93028DE2-DD2C-5C0A-BB63-3F3D9B71D17B

##### Material.

Lebanon: Keserwan-Jbeil Gov.: • 2♂1♀ (ZMUT), Faitroun, 33°59'32"N, 35°44'13"E, 1200 m, 21.4.2022 (C. Reuter).

##### Distribution.

Previously known from Greece, Cyprus, and Turkey. New record for Lebanon, with the present material representing the southernmost known record of the species.

## Discussion

Despite the small size of the collections examined here, and the fact that some consist only of opportunistically collected singleton specimens, a considerable amount of new information was obtained, including six species new to science and numerous new records. This clearly indicates how insufficiently the fauna is currently known. The following taxa were newly recorded from the countries indicated in brackets: *Berlandina* Dalmas, 1922; *B. plumalis* [UAE]; *Coscinida* Simon, 1895; *C. tibialis* [UAE]; *Dorceus* [Saudi Arabia]; *Drassyllus* Chamberlin, 1922; *D. crimeaensis* [Lebanon]; *Episinus*; *E. israeliensis* [Lebanon]; Filistatidae [Saudi Arabia]; *Latrodectus dahli* [Kuwait]; *Leptopilos* [UAE]; *Loureedia* [Saudi Arabia]; *Minosiella* Dalmas, 1921; *M. intermedia* [UAE]; *Nigma* Lehtinen, 1967; *N. conducens* [UAE]; *Oecobius marathaus* [UAE]; *O. putus* [Kuwait, UAE]; *Plexippus paykulli* [Lebanon]; *Sahastata* Benoit, 1968 [Saudi Arabia]; *Synaphosus* [UAE]; *Thanatus imbecillus*; *T. setiger* [Lebanon]; *Thomisus unidentatus*; *T. zyuzini* [UAE]; *Titanoeca* Thorell, 1870; *T. flavicoma* [Lebanon]; *Trichothyse*; *T. golan* [Lebanon]; *Trochosa* C.L. Koch, 1847; *T. dentichelis* [UAE]; *Xysticus kaznakovi* [Lebanon]; *Zelotes* Gistel, 1848; *Z. laetus* [Kuwait, UAE]; *Zodarion barbarae* [Lebanon]. Among the material studied, the record of *N. conducens* represents the easternmost limit of its known range; those of *D. crimeaensis* and *Z. barbarae* represent their southernmost limits; those of *E. israeliensis* and *T. golan* represent their northernmost limits; and that of *T. dentichelis* represents its westernmost limit. Furthermore, the two new eresid species described here extend the known ranges of their respective genera: for *Dorceus*, the easternmost, and for *Loureedia*, the southernmost. In addition, the male of *T. golan* was illustrated and described for the first time.

Of the six new species treated here, *Loureedia melanconi* sp. nov. was described using an integrative approach. The relative simplicity of the copulatory structures in this genus has resulted in extreme similarity of these structures even among geographically distant species that distinctly differ in body pattern (Szűts et al. 2023); consequently, delimiting species of *Loureedia* only on the basis of morphology is generally difficult. With the species described here, seven named species of this genus are currently known; however, both the phylogenetic analysis and the ASAP scoring result selected in this study suggest the presence of two additional cryptic species (Fig. 5C). Furthermore, Shakhatreh et al. (2021) recorded an unidentified male specimen from Zarqa, Jordan, approximately 1200 km in aerial distance from the type locality of *L. melanconi* sp. nov., to which it may belong due to a similar coloration pattern.

Among the regions treated here, no checklists have been published for Lebanon. It is difficult to obtain even a rough estimate of the number of records from this country (as well as from Syria), largely because the historical instability of political borders in the region hampers the assignment of historical records to modern countries (see Zonstein and Marusik 2013). From Kuwait, apart from the present study, only three species have previously been reported: *Halodromus patellidens* (Levy, 1977) (Philodromidae), *Latrodectus cinctus* Blackwall, 1865, and *Oecobius alhoutyae* Wunderlich, 1995 (Wunderlich 1995; Knoflach and van Harten 2002; Muster 2009). In addition, a few scattered records exist in the literature of spiders not identified to species level, belonging to the families Araneidae, Gnaphosidae, Lycosidae, Pholcidae, and Salticidae (Clayton and Pilcher 1983; Al-Khalifa et al. 2012; Al-Mutairi 2025). Although a checklist has been published for the UAE (Feulner and Roobas 2015), it is dominated by species tentatively identified, or identified only to genus level, on the basis of photographs of live individuals, rather than examination of preserved specimens. Consequently, the actual number of species taxonomically documented from the country remains unclear, and a systematic checklist based on verified records is still needed. A checklist of the spiders of Saudi Arabia by El-Hennawy (2014) lists 77 species in 69 genera and 25 families; these numbers have increased only slightly since then, which reflects the continued scarcity of taxonomic surveys in the country (e.g., Zamani and Crews 2019; Zamani et al. 2025b).

## Supplementary Material

XML Treatment for Nigma conducens

XML Treatment for Dorceus saif

XML Treatment for Loureedia melanconi

XML Treatment for Sahastata

XML Treatment for Berlandina plumalis

XML Treatment for Drassyllus crimeaensis

XML Treatment for Leptopilos hajarensis

XML Treatment for Minosiella intermedia

XML Treatment for Synaphosus dulcicola

XML Treatment for Trichothyse golan

XML Treatment for Zelotes laetus

XML Treatment for Nesioneta arabica

XML Treatment for Arctosa

XML Treatment for Arctosa formosa

XML Treatment for Hogna effera

XML Treatment for Trochosa dentichelis

XML Treatment for Wadicosa fidelis

XML Treatment for Oecobius marathaus

XML Treatment for Oecobius putus

XML Treatment for Thanatus imbecillus

XML Treatment for Thanatus setiger

XML Treatment for Prodidomus

XML Treatment for Prodidomus emiratus

XML Treatment for Cyrba ocellata

XML Treatment for Langona pallida

XML Treatment for Phlegra pusilla

XML Treatment for Plexippus paykulli

XML Treatment for Coscinida tibialis

XML Treatment for Episinus israeliensis

XML Treatment for Latrodectus dahli

XML Treatment for Thomisus unidentatus

XML Treatment for Thomisus zyuzini

XML Treatment for Xysticus kaznakovi

XML Treatment for Titanoeca flavicoma

XML Treatment for Zodarion barbarae

## Additional information

### Use of AI

No use of AI was reported.

### Author ORCIDs

Alireza Zamani https://orcid.org/0000-0002-8084-9666

Yuri M. Marusik https://orcid.org/0000-0002-4499-5148

Mostafa R. Sharaf https://orcid.org/0000-0001-8137-0705

Khafiya Al Ketbi https://orcid.org/0009-0007-9511-3567

Tamás Szűts https://orcid.org/0000-0001-8954-0641

### Data availability

All of the data that support the findings of this study are available in the main text.
